# Newborn screening by tandem mass spectrometry for glutaric aciduria type 1: a cost-effectiveness analysis

**DOI:** 10.1186/1750-1172-8-167

**Published:** 2013-10-17

**Authors:** Johannes Pfeil, Stefan Listl, Georg F Hoffmann, Stefan Kölker, Martin Lindner, Peter Burgard

**Affiliations:** 1 Department of General Paediatrics, Division of Inherited Metabolic Diseases, Centre for Paediatric and Adolescent Medicine, University Hospital Heidelberg, Im Neuenheimer Feld 430, Heidelberg 69120, Germany; 2 Department of Conservative Dentistry, University of Heidelberg, Heidelberg, Germany; 3 Munich Center for the Economics of Aging, Max Planck Institute for Social Law and Social Policy, Munich, Germany

**Keywords:** Newborn screening GA-I, Glutaric aciduria type 1, Cost-effectiveness

## Abstract

**Background:**

Glutaric aciduria type I (GA-I) is a rare metabolic disorder caused by inherited deficiency of glutaryl-CoA dehydrogenase. Despite high prognostic relevance of early diagnosis and start of metabolic treatment as well as an additional cost saving potential later in life, only a limited number of countries recommend newborn screening for GA-I. So far only limited data is available enabling health care decision makers to evaluate whether investing into GA-I screening represents value for money. The aim of our study was therefore to assess the cost-effectiveness of newborn screening for GA-I by tandem mass spectrometry (MS/MS) compared to a scenario where GA-I is not included in the MS/MS screening panel.

**Methods:**

We assessed the cost-effectiveness of newborn screening for GA-I against the alternative of not including GA-I in MS/MS screening. A Markov model was developed simulating the clinical course of screened and unscreened newborns within different time horizons of 20 and 70 years. Monte Carlo simulation based probabilistic sensitivity analysis was used to determine the probability of GA-I screening representing a cost-effective therapeutic strategy.

**Results:**

Within a 20 year time horizon, GA-I screening averts approximately 3.7 DALYs (95% CI 2.9 – 4.5) and about one life year is gained (95% CI 0.7 – 1.4) per 100,000 neonates screened initially . Moreover, the screening programme saves a total of around 30,682 Euro (95% CI 14,343 to 49,176 Euro) per 100,000 screened neonates over a 20 year time horizon.

**Conclusion:**

Within the limitations of the present study, extending pre-existing MS/MS newborn screening programmes by GA-I represents a highly cost-effective diagnostic strategy when assessed under conditions comparable to the German health care system.

## Background

Glutaric aciduria type I (GA-I) is a rare genetic disorder with an estimated prevalence of approximately 1:110,000 in Germany [1] and 1:100,000 worldwide [2]. GA-I is caused by inherited deficiency of the mitochondrial enzyme glutaryl-CoA dehydrogenase (GCDH; EC 1.3.99.7) which is involved in the final degradative pathways of the amino acids L-lysine, L-hydroxylysine, and L-tryptophan. The majority of newborns with GA-I are asymptomatic, except for macrocephaly and transient neurological symptoms such as axial hypotonia and asymmetric posturing. Irreversible neurological symptoms generally occur between the age of three months and three years. Symptoms may develop acutely during encephalopathic crises precipitated by catabolism or insidiously without such crises [3]. The underlying cause is irreversible striatal injury resulting in a complex movement disorder with predominant dystonia [4-6]. Previous studies have shown that the outcome is mainly determined by a single crisis during infancy or childhood [3,7].

Several studies have shown that treatment consisting of a low lysine diet, carnitine supplementation, and metabolic emergency treatment during episodes likely to induce catabolism can significantly reduce mortality and morbidity in early diagnosed patients [1,8]. This beneficial therapeutic effect requires early identification of affected newborns, allowing initiation of treatment in a clinically presymptomatic state. Newborn screening for GA-I can be efficiently based on the identification of elevated glutarylcarnitine (C5DC) concentrations in dried blood spots by tandem mass spectrometry (MS/MS). Guidelines have been developed for the diagnosis and management of GA-I [9,10].

Hence, GA-I fulfills major criteria for the inclusion into newborn screening programmes [11]. Considering current national screening panels, GA-I has been recommended as 1 of 29 core panel diseases for newborn screening in the US and was incorporated into nationwide newborn screening in Germany in 2005. A European report published in 2012 indicated that 10 European countries (Austria, Flemish Belgium, Czech Republic, Denmark, Germany, Hungary, Iceland, Netherlands, Portugal and Spain) had included GA-I into their newborn screening panel [12], while 27 countries did not screen for GA-I. Among these 27 European countries, seven countries (Ireland, Luxemburg, Norway, Poland, Sweden, Switzerland and the United Kingdom) were applying MS/MS [13].

A possible reason for this different valuation of newborn screening for GA-I might be scarce information regarding its cost-effectiveness. Several previous studies have analysed the cost effectiveness of introducing expanded MS/MS newborn screening programmes for different selections of diseases, including screening for GA-I [14-17]. A previous review on the cost-effectiveness of neonatal screening concluded that screening for GA-I was likely to be highly cost-effective [18]. At the time this study was performed, clinical data on the effectiveness of early dietary treatment was limited to a small number of studies, and management of the disorder was not clearly defined. In the meantime, several studies have provided further evidence for the effectiveness of early dietary treatment [1,3,19]. To our knowledge, the cost-effectiveness for inclusion of GA-I into pre-existing MS/MS based newborn screening programmes has never been investigated in detail thereafter, even though it is relevant to decision makers who want to offer the best “value for money” to the society [14,20]. The purpose of our analysis is therefore, to determine the cost-effectiveness of including GA-I into MS/MS newborn screening in comparison to the alternative of not including GA-I into the screening panel.

## Methods

### Decision analytic modelling

**Figure 1:**
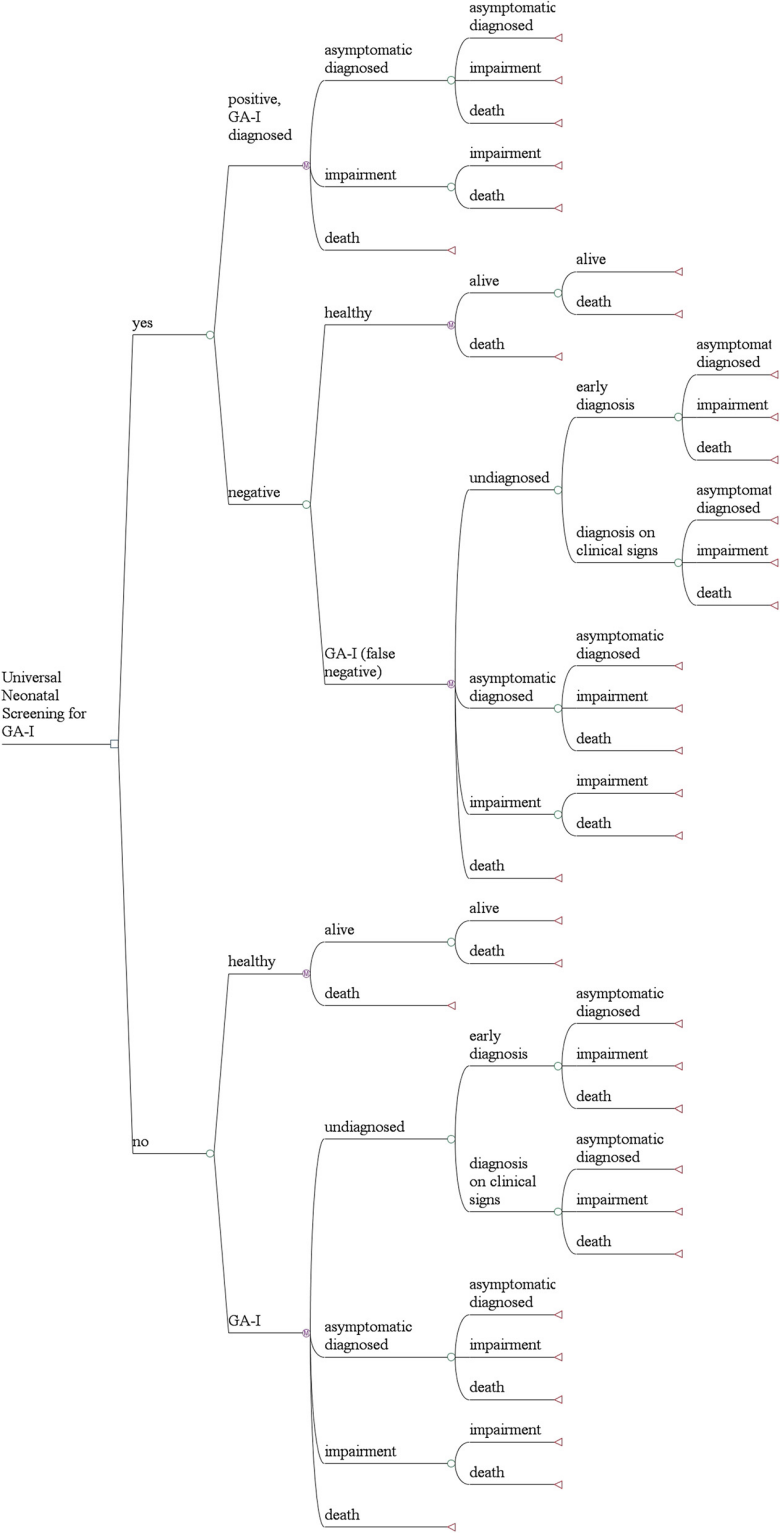
**Markov Model to assess the cost-effectiveness of MS/MS-based neonatal screening for GA-I.** Following the initial screening decision, GA-I can be diagnosed either based on the screening result, based on the clinical sign of macrocephaly (early diagnosis) or following an acute encephalopathic crisis (diagnosis on clinical sign). Regarding the health state, individuals with GA-I can either be healthy or have only mild symptoms (asymptomatic diagnosed), suffer from severe movement disorder (impaired), or have died (death). Healthy individuals with GA-I in whom the diagnosis has not been established (undiagnosed) have a higher risk to experience acute encephalopathic crisis than healthy children with known diagnosis (asymptomatic diagnosed).

We developed a decision-analytic Markov model to evaluate clinical outcomes for hypothetical cohorts of neonates who did or did not undergo screening by MS/MS for GA-I in the newborn period (Figure 1). In health economic analysis, Markov models are a technique to depict probabilities and time durations for cycles in which individuals remain in the same or move on to a different health state [21]. These health states have assigned probabilities, and values for cost and effectiveness. Cost and effectiveness are tallied at the end of each cycle and totalled by the end of the simulation.

We populated our model with published and primary data and simulated the clinical course of a cohort of 100,000 newborns from birth through ages 20 and 70 years. The subjects in the hypothetical cohort were assumed to make annual transitions between a set of health states reflecting clinical status and morbidity. Each health state was associated with an annual cost, an annual effectiveness value, and a set of probabilities for subsequent events. The time spent in each state was used to calculate life expectancy and costs. We adopted a provider perspective and followed standard guidelines of economic analysis [22,23]. Monte-Carlo probabilistic sensitivity analysis [24] was used to assess the probability of the alternative treatment strategies being cost-effective, thereby enabling to determine the robustness of our results. As this study was not experimental and did not include research carried out on humans ethical approval was not necessary.

### Strategies

We modelled two competing scenarios (Figure 1). In the first scenario, newborns do not undergo universal GA-I screening. Individuals either have or do not have the disorder. Unscreened infants without the disorder are and remain unaffected infants. Unscreened infants with the disorder can be in one out of four health states: undiagnosed GA-I, asymptomatic diagnosed GA-I, permanent impairment due to GA-I, or death. “Undiagnosed” represents individuals who have the disorder but who are so far clinically unaffected and thus undiagnosed. “Asymptomatic diagnosed” represents individuals who have the disorder and were diagnosed either due to macrocephaly, by clinical signs including acute encephalopathy or by high-risk screening, but do not suffer from severe permanent impairment defined as severe movement disorder (sMD). Importantly, the “asymptomatic” state also includes persons with milder movement disorders, as these persons usually are not significantly disabled in daily life activities [1]. “Permanent impairment” thus subsumes all individuals who are affected by sMD due to GA-I. This health state is associated with significantly higher cost and higher mortality risk compared to the “asymptomatic” health state. Individuals in the death state have died from either GA-I-related or from other causes.

The second scenario offers newborn screening for GA-I, where neonates can test either positive or negative. We assume that testing is based on current guidelines [10], and that positive newborn screening, including positive confirmatory test results, establishes the diagnosis of GA-I with perfect specificity. However, it is known that false negative screening results occur and thus, baseline analysis included a test sensitivity of 94.5% [1] and was subject to further sensitivity analysis. Following the definitions above, individuals with a confirmed diagnosis of GA-I can be in the health states “asymptomatic”, “impaired” or “death”.

Incremental cost-effectiveness ratios (ICER) were calculated under the assumption that MS/MS had already been introduced for newborn screening. Therefore, only variable costs of adding screening for GA-I are included in the analysis. Our calculations of the true ICER for universal screening for GA-I are conservative as we considered the upper bound when estimating costs, and the lower bound when estimating health outcomes associated with the screening strategy. In the non-screening scenario we considered the lower bound of costs and the upper bound of health outcomes.

Thus, the reported cost-effectiveness ratios for universal neonatal GA-I screening are likely to be upper-end estimates, following standard guidelines of economic cost-effectiveness analysis [22,25]. Cost and effectiveness were discounted at an annual rate of 3% and calculated for two time horizons of either 20 or 70 years, in accordance with the time horizon considered in a previously published cost-effectiveness analysis for medium-chain acyl-CoA dehydrogenase deficiency screening [26].

### Data sources

Data and data sources used in the model are shown in Table 1. Sources of data included previous publications on the outcome in GA-I following either clinical diagnosis or newborn screening. Data was supplemented by primary data of a 10-year prospective follow-up study performed at our study centre [27], as well as expert clinical opinion from the authors SK and ML (assumptions).

**Table 1:** Effectiveness and cost input variables used in the analysis

	**Distribution**	**Distribution parameters**	**Data source**
**Epidemiology, prevalence and effectiveness**			**Base case**
Test Specificity (including confirmation by enzyme analysis)	Point estimate	1	Assumption
Test Sensitivity	Triangular	Mode 0.945 (min-max 0.9-0.99)	[1,3]
Prevalence based on positive test result	Triangular	Mode: 1 in 112,700, (min-max 1 in 129,455 – 1 in 95,953)	[1]
Probability of early diagnosis before the onset of sMD	Triangular	Mode 0.15 (min-max 0.12-0.18)	[3]
Probability that severe movement disorder develops in previously diagnosed children	Triangular	Mode 0.115 (min-max 0.092-0.138)	[1]
Probability that severe movement disorder occur following clinical manifestation	Triangular	Mode 0.74 (min-max 0.592-0.888)	[28]
Life expectancy of healthy population	Point estimate	79.45 years	[29]
Life expectancy in asymptomatic persons with GA-I	Equal to life expectancy of healthy population		Assumption
Life expectancy in impaired health state	Uniform	Min-max 25–45 years	[3]
**Cost Assessment**			
**A) Direct screening cost, including genetic and enzymatic confirmation studies**			
Initial medical cost for children experiencing an acute encephalopathic crisis	Triangular	Mode 3000 (min-max 2000–8000)	Assumption, based on year 2010 German DRG reimbursement
Cost for genetic and enzymatic confirmation studies	Point estimate	865	Primary data
Direct screening cost per neonate	Point estimate	0.031	Calculated, based on [30]
**B) Cost for medical and dietary treatment in persons with GA-l**			
Cost per outpatient visit	Triangular	Mode 127 (min-max 88.9 -165.1)	Primary data
Cost per inpatient treatment	Triangular	Mode 1335 (min-max 934.5 - 1735.5)	Primary data
Number of outpatient treatments per year	Point estimate	*Age-depending*	[10]
Number of inpatient treatments per year	Point estimate	*Age-depending*	[10]
Cost for basic dietary treatment per year	Point estimate	*Age-depending*	[10]
**C) Cost associated with severe movement disorder**			
Annual cost for special schooling (included for age 6 to 16)	Triangular	Mode 5689 (min-max 0–11369)	Assumption, based on [31]
Annual cost for special care, starting after age of 6	Triangular	Mode 2700 (min-max 1890–3510)	Assumption, based on [32]
Annual overhead cost in case of severe movement disorder	Uniform	Min-max 0-3000	Assumption

All costs are expressed in Euro for the year 2010.

### Course of disease

If newborn screening for GA-I is not established, GA-I patients can be diagnosed either early due to clinical signs like macrocephaly, a known index case in an affected family, or after the manifestation of irreversible neurologic symptoms (e.g. complex movement disorder).

In the scenario without newborn screening for GA-I, we assumed that diagnosis based on high-risk screening of macrocephaly is established in 15% of cases, while the other 85% of cases will be diagnosed after clinical presentation. These figures were derived from a previous report of a total of 279 patients. Among these, 23 were diagnosed by neonatal screening while of the remaining 256 individuals, 24 were diagnosed by high-risk screening, 14 by macrocephaly (combined 38/256 = 14.8%) and 218 individuals (218/256 = 85.2%) were detected after clinical presentation [3].

For infants being diagnosed early on the basis of high-risk screening, we assumed the clinical course to be identical to the cohort undergoing newborn screening. In cases where diagnosis is made following (non-macrocephalic) clinical presentation, we assumed 74% to suffer from sMD [28].

The scenario presenting a universal neonatal screening for GA-I includes 11.5% of the diagnosed cases to suffer from sMD. Even though consequent basic and emergency treatment may lead to a better outcome, 11.5% is a conservative estimate regarding the possibility of insufficient treatment as previously reported [1]. For false negative screening results, the course of disease was assumed to be equal to the outcome of the previously described strategy without universal neonatal screening.

Mortality in the asymptomatic health state was assumed to be equal to the healthy population as reported in the official German mortality table in 2009 [29].

For children affected by severe movement disorder, it has been reported that as many as 50% die before the age of 25 years, often due to severe aspiration pneumonia [3]. Considering likely improvement of current treatment options, we estimated a mean life expectancy of 35 years for individuals in the “impaired” health state for base case analysis and included this estimate into sensitivity analysis.

### Cost assessment

The GA-I screening process includes the initial MS/MS-based newborn screening cost (direct screening cost), and in case of a positive test result confirmation testing, including metabolites, enzyme activity and mutation analysis.

We considered a scenario where MS/MS had already been established for newborn screening. In this case, extension of the pre-existing screening by GA-I will cause only minimal additional cost, since C5DC is only one of many acylcarnitines which are detectable using the same analytical process, i.e. MS/MS. We conservatively assumed that the inclusion of GA-I would lead to an additional recall rate of 0.08%, corresponding to the total recall rate in the German screening panel for all screened diseases by MS/MS with exclusion of phenylketonuria and medium chain acyl-dehydrogenase deficiency. A recall will cause a cost of 31.01 Euro for clinical examination of the newborn and taking a new blood sample by a physician plus 7.00 Euro for repeated MS/MS analysis [33].

We thereby calculated that inclusion of GA-I into the already established MS/MS-based newborn screening panel would result in an additional cost of 0.031 Euro per neonate. However, this value only reflects the cost of GA-I screening for a scenario with a pre-existing MS/MS newborn screening programme. In a situation where MS/MS screening is performed only for a limited number of target conditions, inclusion of GA-I in the screening panel might necessitate resourcing of additional internal standards for acylcarnitines and thereby cause additional screening costs. Furthermore, in a scenario where newborn screening is based on another (non MS/MS) screening technology, inclusion of GA-I into the screening panel will require the introduction of MS/MS technology, possibly resulting in a considerably higher marginal screening cost per newborn. We applied one-way sensitivity analysis to assess the influence of screening cost on the ICER of GA-I newborn screening.

Following a positive screening result, the diagnosis of GA-I is confirmed by metabolite, mutation analysis and/or enzyme activity. The cost of these tests is 25, 800 and 800 Euro, respectively. In current practice, mutation analysis is usually done prior to enzyme activity. Mutation analysis has a sensitivity of around 98%, and only in unclear cases enzyme activity is determined. For this study, we assume that enzyme activity is required in 5% of children with a positive MS/MS screening result, leading to a total cost of 865 (=25+800+0.05*800) Euro.

For children in whom the diagnosis of GA-I is based on clinical symptoms (complex movement disorder with predominant dystonia), we estimated a total cost of 3,000 Euro, including possible inpatient treatment and confirmation analysis. This assumption might underestimate the true costs as some children will probably undergo several tests and hospital stays before the diagnosis can be made, but is in line with the conservative assumption for the cost of the scenario without universal newborn screening.

We further assumed that all infants diagnosed with GA-I receive treatment according to current guidelines [10]. This dietary treatment includes a low lysine diet using natural food with low lysine content and lysine-free amino acid supplements during the first six years of life. The amount of lysine-free amino acid supplements is dependent on the individual diet. We assumed that on average, 40% of the daily amino acid intake would be covered by lysine-free amino acid supplements. We additionally considered the cost of levocarnitine substitution (100 mg/kg/d for the first six years of life, followed by 30 mg/kg/d for lifetime).

In addition to dietary treatment, the cost for out- and inpatient treatment due to GA-I was accounted for in all diagnosed persons. The frequency of such interventions was based on current treatment recommendations [10], while the cost per in- or outpatient treatment was calculated on the basis of the general diagnosis-related group (DRG) reimbursement system in Germany in the year 2010.

For children affected by sMD, additional costs including special care and schooling were considered in the model. It is very likely that severely affected individuals will require a higher frequency of medical interventions. However, we did not have sufficient data to assess these costs as only very few children in our study cohort suffer from severe movement disorder. Thus, in our model the same frequency of in- and outpatient treatment was assigned to individuals in the impaired health state and asymptomatic individuals. As this approach is very likely to underestimate the true costs in the impaired health state, sensitivity analysis included analysis of an overhead lump sum assigned to the impaired state. Table 1 shows key input parameters of the model. All costs were calculated in Euros for the year 2010.

### Effectiveness

The primary measure of clinical outcome was the number of Disability Adjusted Life Years (DALYs) in each of the treatment arms. DALYs is a measurement of overall disease burden, combining the expected years of life lost because of reduced life expectancy with years lived with disability [34]. In our analysis, using DALYs as primary measure of screening effectiveness thereby accounts for both, life years lost due to early mortality as well as for life years with disability caused by sMD. In general, one DALY is equal to one year of healthy life lost. In order to calculate DALYs, disability weights are used to reflect the severity of a health state on a scale from 0 (perfect health) to 1 (equivalent to death). In the absence of an established disability weight for children suffering from sMD, we had to make an estimate on a disability weight that could adequately reflect the impact of severe sMD. Among the disability weights for diseases and conditions published by the WHO [35,36], we found that the disability weight for mental retardation following meningitis (disability weight of 0.459, range 0.402 to 0.484) and the disability weight for neurological sequelae following cerebral malaria (0.471, range 0.443 to 0.471) might adequately reflect the impact of sMD. Common features of neurological sequelae following cerebral malaria in children include hemiplegia, cortical blindness, aphasia and ataxia [37], while hearing loss, mental retardation, motor abnormalities and seizures are common neurological sequelae following bacterial meningitis [38]. For our primary analysis, we approximated the impact of sMD by using the published disability weight of neurological sequelae following malaria infection.

As secondary effectiveness measure, we considered the number of average life years gained when comparing screening vs. not screening. The sum of the annual cycles spent in all states other than death represented average life expectancy of members of the cohort. Age weighting was not applied, thus a year of healthy life was valued equally at all ages.

### Sensitivity analysis

To assess the uncertainty in the model and the robustness of our results, we used one-way sensitivity analysis and Monte Carlo probabilistic sensitivity analysis. One-way sensitivity analysis assesses the influence of single parameters on a result. Monte-Carlo probabilistic sensitivity analysis (50,000 repetitions) simultaneously varied input variables over their full range (Table 1).

## Results and discussion

Table 2 shows the predicted effectiveness of newborn screening for GA-I. For a 20 years horizon, the screening programme averts approximately 3.7 DALYs (95% CI 2.9 – 4.5) and about 1.0 life year will be gained (95% CI 0.7 – 1.4) per 100,000 neonates screened initially. When followed for 70 years, 4.1 life years (95% CI 3.0 – 5.7) in a cohort of 100,000 screened neonates and a total of 6.0 DALYs (95% CI 4.7 – 7.5) are averted by GA-I newborn screening.

**Table 2:** Predicted effectiveness of the screening programme per 100,000 neonates

**20 years horizon**	**Mean value**	**95% CI**
Life years gained	1.0	0.7 – 1.4
DALY averted	3.7	2.9 – 4.5
**70 years horizon**	**Mean value**	**95% CI**
Life years gained	4.1	3.0 – 5.7
DALY averted	6.0	4.7 – 7.5

Extension of a pre-existing MS/MS newborn screening programme by GA-I saves a total of approximately 30,682 Euro (95% CI 14,343 to 49,176) and 36,743 Euro (95% CI 20,178 to 58,531) per 100,000 screened neonates over the 20 and 70 year time horizon, respectively (Table 3). Thus, the strategy with newborn screening for GA-I is more effective, as well as cost saving compared to the strategy without screening for a 20, as well as for a 70 year horizon.

**Table 3:** Incremental cost of universal GA-I newborn screening per 100,000 neonates

	**Mean value**	**95% CI**
Incremental cost, 20 years horizon	−30,682 €	−14,550 € to −49,401 €
Incremental cost, 70 years horizon	−36,743 €	−19,072 € to −57,365 €

One-way sensitivity analysis was used to further assess the influence of single input parameters on the model outcome. Input parameters were assessed according to Table 1, and all results showed that the strategy with newborn screening remained the dominant, i.e. cost-saving intervention (data not shown).

**Figure 2:**
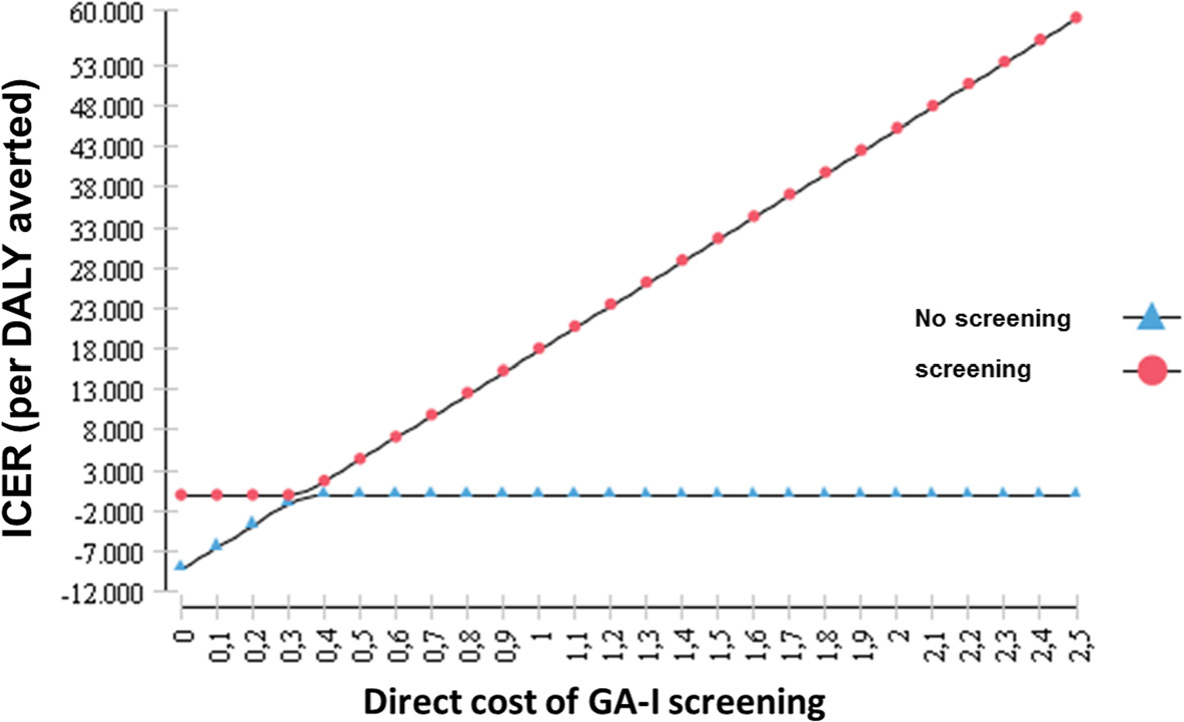
ICER (In EURO per DALY averted) depending on the initial cost of GA-I screening (direct cost of GA-I screening, in EURO per newborn over a 20 year horizon).

As GA-I is a rare metabolic disorder, we expected that the cost for the initial screening, which is attributable to all newborns, would be a critical parameter in the cost-effectiveness evaluation of the GA-I screening programme. This is of special importance as the incremental cost of neonatal GA-I screening is dependent on the screening programme already available. Our analysis considers a scenario where MS/MS screening for the whole range of acylcarnitines is already established and extended to a wider range of target conditions. In such a scenario, the incremental cost of GA-I screening might be negligible. In contrast, in a scenario where MS/MS screening is pre-established but includes only a limited number of target conditions (e.g. representing the current screening practice in the UK), inclusion of GA-I in the screening panel might require the introduction of additional internal standards for acylcarnitines and would thereby result in an incremental cost of about 0.2 to 0.3 Euros per neonate [39]. Figure 2 illustrates the ICER of the screening versus the non-screening scenario at different values of the initial screening cost at a 20 years horizon. Inclusion of GA-I into the newborn screening panel was found to be cost saving if the screening cost was below 0.34 Euro per initially screened neonate. At a higher screening cost, the strategy with GA-I screening is expected to be more expensive than the strategy without screening. At a cost of e.g. 1.07 Euro per neonate, GA-I screening results in an incremental cost-effectiveness of 20,000 Euro per DALY averted. Thus, inclusion of GA-I screening into a pre-existing MS/MS based screening panel remains a cost saving intervention even in a scenario where additional internal standards for acylcarnitines are required. For other countries widely differing prevalence rates for GA-I, ranging from 1 in 35000 in Spain [40] to 1 in 354000 in California [41] have been reported. We assessed the impact of these prevalence rates on cost and cost-effectiveness estimates in an independent analysis for a 20 years horizon at incremental screening costs of 0.03 and 0.3 Euros per neonate, reflecting a scenario with or without pre-established GA-I acylcarnitine internal standards, respectively.

At 0.03 Euro incremental screening cost, extension of an existing MS/MS screening programme by GA-I remains a cost-saving intervention at any prevalence assessed. Assuming an incremental screening cost of 0.3 Euro per neonate, the intervention ceases to be cost-saving at a prevalence of 1 in 200,000, with a resulting ICER of approximately 5,700 Euro (95% CI 651 – 10,728) per DALY averted (Table 4). It is important to note, that these findings are based on the cost assumptions reported in Table 1.

**Table 4:** Cost and cost-effectiveness of universal GA-I newborn screening at differing prevalence rates and low versus high incremental screening cost

	**Incremental screening cost 0.03 Euro/neonate**		**Incremental screening cost 0.3 Euro/neonate**	
**Prevalence**	**Mean incremental cost per 100000 screened neonates (95% CI)**	**ICER**	**Mean incremental cost per 100000 screened neonates (95% CI)**	**ICER (95% CI)**
**1 in 35,000**	−103,445 (−160,339 – -52,919)	<0	−76,545 (−133,439 – -26,019)	<0
**1 in 100,000**	−34,191 (−54,104 – -16,507)	<0	−7,291 (−27,204 – 10,393)	−1,750 (−6,417 - 2,657)
**1 in 200,000**	−15,545 (−25,502 – -6,703)	<0	11,355 (1,398 – 20,197)	5,703 (651 – 10,728)
**1 in 350,000**	−7,555 (− 13,244 – - 2,502)	<0	19,345 (13,656 – 24,398)	16,882 (10,800 – 23,491)

All values in Euro, 20 years horizon.

The WHO Commission on Macroeconomics and Health classified interventions as highly cost effective if the cost per DALY averted was less than the gross domestic product (GDP) per head, and as cost effective if this cost was less than one-to-three times the GDP per head. With the 2010 per capita GDP of $ 38,170 for Germany and $ 34,177 for the Euro area (adjusted for purchasing power parity and expressed in international dollars) as a threshold [42], inclusion of GA-I into MS/MS newborn screening programmes is a highly cost effective health intervention according to our study results.

In a scenario without pre-existing MS/MS newborn screening, introduction of GA-I screening necessitates a change of screening technology. In this case, the incremental cost of GA-I screening might widely differ from our baseline assumptions and furthermore, not only GA-I, but also other conditions, even those hitherto screened for by other methodology, could be included in the screening panel with the change of screening methodology. Our analysis was not designed to assess the economic consequences of such a scenario. Several previous studies have evaluated the cost-effectiveness of introducing MS/MS newborn screening, reporting that MS/MS screening for a combination of different diseases is attractive from an economic perspective [15-18,43].

Regarding generalizability of our findings, some concern may be that these results would only be valid under the conditions of the German health system. This applies especially to the estimation of cost, as the precise costs we incorporated in our decision analytic model can, of course, only be considered fully reliable for the specific scenario assumed. In contrast, the clinical evidence that our study is based on is not restricted to a single geographic setting but is derived from a variety of European countries and Australia [1,7,28,44,45].

Regarding the estimation of cost, sensitivity analysis showed that, with exception of the initial screening cost, our results are robust for all relevant input parameters and the scenario with universal GA-I screening remained cost saving in all analyses at a 20, as well as a 70 year horizon. Therefore, it is reasonable to assume that inclusion of GA-I into the newborn screening panel will be a highly cost-effective health intervention not only in Germany, but also in many other countries with similar health and welfare systems.

We used DALYs as primary outcome measure, while recent health economic evaluations of neonatal medium chain acyl-CoA dehydrogenase deficiency (MCADD) reported quality adjusted life years (QALYs) [46,47]. Both, QALYs and DALYs, are measures of disease burden allowing to combine morbidity and mortality in a single outcome measure [48]. QALYs are derived by assessment of health states using different methods like time-trade-off, visual analogue scales or standard descriptive systems like the EuroQuol EQ5D questionnaire [49]. In contrast, DALYs rely on disability weights associated with specific diseases (rather than health states), and they are usually derived through expert opinion. Groups of experts rated the severity of disabling sequelae initially according to six disability classes [34] or in a revised version by 22 indicator conditions [50]. It is evident that all available outcome measures for health economic evaluations have their strengths and limitations [48,51]. Regarding the DALY concept, major points of criticism are the exclusion of the patient’s health perception as well as the living circumstances of individuals who experience morbidity [52]. In contrast, regarding QALYs, it has been discussed that measuring the health status of children raises important methodological issues like the age-dependency of health perception as well as the influence of parents [53]. Moreover, the QALY concept has been criticised for an overall lack of quality in paediatric care [54]. Regarding the present analysis, no suitable data was available for calculating QALYs according to methodological standards. Furthermore, self-assessment or the use of time-trade-off techniques seems unfeasible in children suffering from severe impairments [55]. We therefore made an expert assumption among the study authors to derive an appropriate disability weight for our analysis. We felt that the evidence level is best reflected by using DALYs for health outcome measurement. Despite the fact that there may always be additional information gained from health economic evaluation, it is pivotal to be aware of the limitations implied by the particular method used, especially when only one specific health outcome measure (e.g. DALYs) is feasible for application. As there is no well-established disability weighting for inherited metabolic disorders, cost-effectiveness analysis should only be one among several aspects in decision making. For future health economic assessment, it would be desirable to establish a broader consensus on appropriate outcome measurement.

A final general concern regarding our analysis is the extent and quality of available evidence which our analysis is based on. Current knowledge of the clinical course of GA-I is based on a relatively small number of studies [1,7,28,44,45]. In addition, sample sizes of these studies may be regarded as comparably small. Therefore, our results indicate considerable uncertainty and should thus be interpreted with caution. It is of special concern that the natural course and outcome of screened and unscreened individuals with GA-I was derived from previously published studies [1,3,28]. In these historical cohorts, unscreened individuals were born earlier, i.e. before introduction of MS/MS based screening for GA-I. Therefore, the superior outcome of screened individuals might not only be attributable to the screening itself, but also to other, time-dependant improvements in health care. In contrast, it is possible that prior to the introduction of newborn screening for GA-I, a significant amount of children suffering from GA-I might have deceased without the diagnosis being established. Thus, the reported data might both, over- as well as underestimate GA-I related morbidity and mortality in unscreened individuals.

The availability of limited clinical data is a constraint which applies to any rare disease. In these situations the only choice is to base decisions about cost-effectiveness on the currently available evidence and to use uncertainties for determining domains where further research is needed [56]. Introduction of large-scale, multi-national databases for patients with rare metabolic diseases might be a useful tool to provide more detailed information on the clinical course of patients with rare metabolic disorders, e.g. the European registry and network for intoxication type metabolic disorders [56] (https://www.eimd-registry.org/).

## Conclusion

In line with current evidence, our findings indicate that the morbidity resulting from GA-I can be considerably reduced by early diagnosis and treatment. Moreover, the long-term costs of screening for GA-I were shown to be lower than excluding GA-I from a MS/MS screening panel. To extend a pre-existing MS/MS screening by GA-I is a cost saving health intervention under conditions comparable to the German health system. Our findings may also apply to other countries with similar health and welfare systems. The inclusion of GA-I into newborn screening programmes merits serious consideration by health policy makers.

### Availability of supporting data

The data sets supporting the results of this article are included within the article.

## Abbreviations

DALY: Disability adjusted life year; GA-I: Glutaric aciduria type 1; GDP: Gross domestic product; ICER: Incremental cost-effectiveness ratio; MS/MS: Electrospray-ionization tandem mass spectrometry; sMD: Severe movement disorder; WHO: World Health Organisation.

## Competing interests

The authors declare that they have no competing interests.

## Authors’ contributions

The decision analytical model was conceived by JP, SL and PB. Model analysis and data interpretation was done by JP and PB. GFH, SK and ML provided expert clinical opinion and substantial input for data analysis. JP, SL and PB wrote the manuscript. All authors read and approved the final manuscript.
